# Intravenous thrombolysis in acute ischemic stroke after antagonization of unfractionated heparin with protamine: case series and systematic review of literature

**DOI:** 10.1177/17562864221149249

**Published:** 2023-01-24

**Authors:** Katharina Kneer, Adedolapo Kamaldeen Adeyemi, Jennifer Sartor-Pfeiffer, Vera Wilke, Corinna Blum, Ulf Ziemann, Sven Poli, Annerose Mengel, Katharina Feil

**Affiliations:** Centre for Neurovascular Diseases Tübingen (ZNET), Tübingen, Germany; Department of Neurology and Epileptology, Eberhard Karl University of Tübingen, Tübingen, Germany; Hertie Institute for Clinical Brain Research, Tübingen, Germany; Department of Neurology & Stroke, Eberhard Karl University of Tübingen, Tübingen, Germany; Department of Neurology & Stroke, Eberhard Karl University of Tübingen, Tübingen, Germany; Department of Neurology & Stroke, Eberhard Karl University of Tübingen, Tübingen, Germany; Department of Neurology & Stroke, Eberhard Karl University of Tübingen, Tübingen, Germany; Centre for Neurovascular Diseases Tübingen (ZNET), Tübingen, Germany; Department of Neurology & Stroke, Eberhard Karl University of Tübingen, Tübingen, Germany; Hertie Institute for Clinical Brain Research, Tübingen, Germany; Centre for Neurovascular Diseases Tübingen (ZNET), Tübingen, Germany; Department of Neurology & Stroke, Eberhard Karl University of Tübingen, Tübingen, Germany; Hertie Institute for Clinical Brain Research, Tübingen, Germany; Centre for Neurovascular Diseases Tübingen (ZNET), Tübingen, Germany; Department of Neurology & Stroke, Eberhard Karl University of Tübingen, Tübingen, Germany; Hertie Institute for Clinical Brain Research, Tübingen, Germany; Department of Neurology & Stroke, Eberhard Karl University of Tübingen, Hopple-Seyler- Strasse 3, 72076 Tübingen, Germany; Centre for Neurovascular Diseases Tübingen (ZNET), Tübingen, Germany; Hertie Institute for Clinical Brain Research, Tübingen, Germany

**Keywords:** acute ischemic stroke, antagonization, heparin, intravenous thrombolysis, protamine, revascularization

## Abstract

**Background and aims:**

Intravenous thrombolysis (IVT) is standard of care for disabling acute ischemic stroke (AIS) within a time window of ⩽ 4.5 h. Some AIS patients cannot be treated with IVT due to limiting contraindications, including heparin usage in an anticoagulating dose within the past 24 h or an elevated activated prothrombin time (aPTT) > 15 s. Protamine is a potent antidote to unfractionated heparin.

**Objectives:**

The objective of this study was to investigate the safety and efficacy of IVT in AIS patients after antagonization of unfractionated heparin with protamine.

**Methods:**

Patients from our stroke center (between January 2015 and September 2021) treated with IVT after heparin antagonization with protamine were analyzed. National Institutes of Health Stroke Scale (NIHSS) was used for stroke severity and modified Rankin Scale (mRS) for outcome assessment. Substantial neurological improvement was defined as the difference between admission and discharge NIHSS of ⩾8 or discharge NIHSS of ⩽1. Good outcome at follow-up after 3 months was defined as mRS 0–2. Safety data were obtained for mortality, symptomatic intracerebral hemorrhage (sICH), and for adverse events due to protamine. Second, a systematic review was performed searching PubMed and Scopus for studies and case reviews presenting AIS patients treated with IVT after heparin antagonization with protamine. The search was limited from January 1, 2011 to September 29, 2021. Furthermore, we conducted a propensity score matching comparing protamine-treated patients to a control IVT group without protamine (ratio 2:1, match tolerance 0.2).

**Results:**

A total of 16 patients, 5 treated in our hospital and 11 from literature, [65.2 ± 13.1 years, 37.5% female, median premorbid mRS (pmRS) 1 (IQR 1, 4)] treated with IVT after heparin antagonization using protamine were included and compared to 31 IVT patients [76.2 ± 10.9 years, 45% female, median pmRS 1 (IQR 0, 2)]. Substantial neurological improvement was evident in 68.8% of protamine-treated patients *versus* 38.7% of control patients (*p* = 0.028). Good clinical outcome at follow-up was observed in 56.3% *versus* 58.1% of patients (*p* = 0.576). No adverse events due to protamine were reported, one patient suffered sICH after secondary endovascular thrombectomy of large vessel occlusion. Mortality was 6.3% *versus* 22.6% (*p* = 0.236).

**Conclusion:**

IVT after heparin antagonization with protamine seems to be safe and, prospectively, may extend the number of AIS patients who can benefit from reperfusion treatment using IVT. Further prospective registry trials would be helpful to further investigate the clinical applicability of heparin antagonization.

## Introduction

Intravenous thrombolysis (IVT) with recombinant tissue plasminogen activator (rtPA) is standard of care for acute ischemic stroke (AIS) with disabling symptoms within a time window of 4.5 h after stroke symptom onset.^1,2^ However, not all AIS patients are eligible for IVT as the total number of patients receiving IVT is limited due to absolute and relative contraindications.^3^ However, 64.8% of all AIS patients receiving IVT fulfill at least one of the absolute or relative contraindications.^3–6^ Ongoing anticoagulation including unfractionated heparin (UFH), non-vitamin K antagonist oral anticoagulants (NOAC), and vitamin K antagonists (VKA) are known restrictions for IVT.^7,8^ Regarding the eligibility criteria for the treatment of AIS with IVT, specifically therapeutic doses of low-molecular weight heparin (LMWH) received within 24 h are a substantial exclusion criterion. This exclusion does not apply to prophylactic doses.^1,9^ Up to 17% of all strokes occur during hospital admission, with in-hospital strokes being one of the most common complications during a hospital stay with a total incidence of 0.06%.^10^ Especially, cardiac surgery and percutaneous interventions are associated with a relevant rate of peri-interventional strokes due to mechanical manipulation, hypotension during the procedures, or cardiac arrhythmias, with a total risk of approximately 0.7–7%.^11,12^ These procedures often require administration of UFH. Therefore, patients experiencing AIS during or shortly after the intervention cannot receive IVT due to the use of heparin. UFH has a half-life of 60–150 min when administered at higher dosage (100 and 400 IU/kg), and of 30 min when administered at lower dosage (25 IU/kg).^13^

Protamine is a specific antagonist to heparin by binding heparin and forming a heparin–protamine salt structure within 30 min, which *per se* leads to an ineffective state and is then further metabolized and excreted. The exact mechanism of action remains unknown.^14–18^ Protamine is a cheap and accessible antidote to heparin, does not increase the risk of thromboembolism^16–20^ and yet, only limited data are available describing the antagonization of heparin before initiating IVT.^21^

The aim of our study was to systematically analyze AIS patients treated with IVT after antagonization of heparin using protamine to assess the feasibility, safety, and the clinical outcome of IVT treatment in these patients.

## Methods

### Case series, study population

All consecutive patients between January 2015 and September 2021 treated with IVT in our comprehensive stroke center at the University hospital of Tübingen (UKT) were retrospectively collected and analyzed. All patients who were treated with IVT after heparin antagonization with protamine were included. The activated prothrombin time (aPTT) was used to estimate treatment efficacy of heparin. A successful antagonization of heparin action with protamine was identified by aPTT normalization to < 25 s, according to our laboratory cut-off (upper value > 120 s, lowest value 18 s). Treatment decisions were made by experienced neurologists and based on clinical and imaging parameters according to national and international guidelines.^1,9^

Stroke severity was assessed by the National Institutes of Health Stroke Scale (NIHSS), a scoring system which is used to rate the neurological deficit of the patient ranging from 0 to 42 points, with higher points indicating more severe deficits.^22^ Substantial neurological improvement was defined as the difference between admission and discharge NIHSS of ⩾ 8 or discharge NIHSS of ⩽ 1, as described previously.^23,24^ The degree of dependence or disability was rated by the modified Rankin Scale (mRS) and the premorbid mRS (pmRS).^25^ Functional outcome was assessed using the mRS either by phone calls or outpatient visits. Clinical outcome was defined *good* if mRS was 0–2 and *excellent* if mRS was 0–1 according to European Stroke Organization (ESO) definition (https://eso-stroke.org/outcome-measures-stroke-modified-rankin-scale-ordinal-logistic-regression/).^26–28^ Early signs of infarction on imaging were assessed using the Alberta Stroke Program Early CT Score (ASPECTS).^29^

Symptomatic intracerebral hemorrhage (sICH) was defined according to ECASS-3 (any hemorrhage with neurological deterioration as indicated by an NIHSS that was 4 points higher than at baseline or the lowest value in the first 7 days, or any hemorrhage leading to death; in addition, the hemorrhage must have been identified as the predominant cause of the neurologic deterioration).^30^ Furthermore, the incidence of adverse events due to protamine was assessed.

### Review: search strategy and inclusion criteria

**Figure 1.**
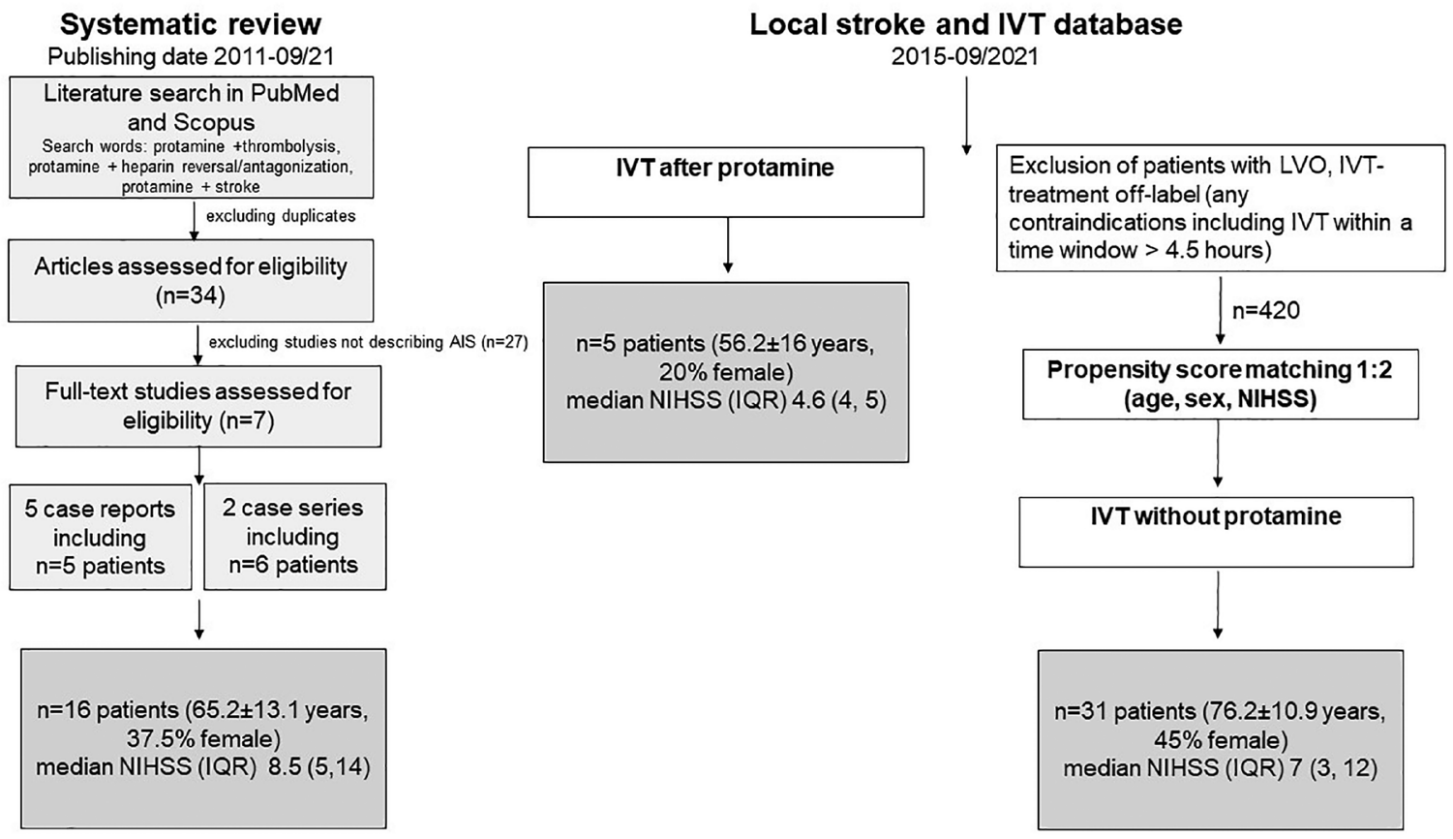
Flow chart of literature review with inclusion and exclusion of studies/case reports and of case series. AIS, acute ischemic stroke; IQR, interquartile range; IVT, intravenous thrombolysis; n, number; NIHSS, National Institutes of Health Stroke Scale; SD, standard deviation.

We searched PubMed and Scopus for studies and case reviews presenting AIS patients treated with IVT after heparin antagonization with protamine sulfate using the terms: ‘Protamine and heparin reversal’, ‘Protamine and antagonization’, ‘protamine and thrombolysis’, or ‘protamine and stroke’. The search was limited from January 1, 2011 to September 29, 2021. In addition, references were screened for related letters, reviews, and editorials to include further eligible studies (see Figure 1).

### Outcomes and data extraction

Our outcomes were defined by sICH, pmRS, mRS at discharge, and changes in NIHSS after the administration of thrombolysis. Computed tomography (CT) or magnetic resonance imaging (MRI) was performed for each patient 24 h after IVT to assess sICH. Additional cerebral imaging was performed in case of neurological deterioration.

### Statistical analysis

Data were collected using Excel (Microsoft, Redmond, WA) spreadsheet software. Continuous variables were tested for normal distribution using the Kolmogorov–Smirnov test. Normally distributed data were presented as mean and standard deviation (± SD) and non-normally distributed data as median with interquartile range (IQR, 25% and 75% percentile). We further conducted a propensity score matching (PSM) to compare patients treated with IVT after protamine and without protamine. The control group was randomly selected from our patients during the same period using propensity scores to match patients with stroke treated with IVT after protamine and without protamine within a time window after known symptom onset of 4.5 h for age, sex, and NIHSS. We used the optimal method with a ratio of 1:2 and a match tolerance of 0.2. Between the groups, comparisons in baseline characteristics were assessed using univariate generalized models (binomial or multinomial distribution) for categorical variables and linear mixed model with matched sets as random effect for quantitative variables. We used binomial distribution and logit function to compare outcome variables. For all statistical testing, we used the Statistical Package for Social Science (SPSS for Windows v27.0, IBM).

## Results

In total, five patients [56.2 ± 16.0 years, 20% female, median pmRS 1 (IQR 2, 5)] were treated with IVT after antagonization of heparin using protamine in our clinic. Heparin was given within the last 6 h before stroke symptom onset. One patient received heparin for persistent foramen ovale occlusion and four during percutaneous cardiac catheterization. Protamine was given prior to IVT to antagonize heparin depending on heparin dosage (see Table 1). The median time from stroke symptom onset to IVT treatment was 120 min (IQR 60, 180), the median time of protamine administration prior to IVT was 28 min (IQR 2.5, 65.5). However, 80% of our patients improved clinically regarding NIHSS with a median NIHSS shift of 3 points on discharge. Neither systemic bleeding complications nor sICH occurred. Furthermore, no adverse events due to protamine were reported.

**Table 1.** Baseline characteristics of patients treated with IVT after antagonization of UFH with protamine.

Case	Sex	Age (years)	Heparin dosage (IU)	Protamine dosage (IU)	PTT (s) before protamine	PTT (s) after protamine	IVT dosage (mg/kg)
Case 1	M	56	8000	8000	51	18.0	0.9
Case 2	F	65	25,000/24 h	8000	59	18.0	0.6
Case 3	M	79	5000	5000	24	24.0	0.9
Case 4	M	51	5000	5000	24	21.0	0.9
Case 5	M	30	7500	7500	>160	22.0	0.9
Case 6 Fontaine *et al.*^31^	M	73	7000	4000	110	26.0	0.9
Case 7 Bereczki *et al.*^32^	M	70	Enoxaparin 1 mg/kg s.c. bd	6000	N/A	N/A	0.9
Case 8 Warner *et al.*^12^	M	87	13,000	4000	181	‘normalized’	0.6
Case 9 Safouris *et al.*^33^	M	66	N/A	N/A	59	35.0	0.9
Case 10 Danoun *et al.*^34^	F	52	5000	2500	51.4	25.0	0.9
Case 11 Guevara *et al.*^35^	F	68	5000	5000	N/A	26.1	0.6
Case 12 Guevara *et al.*^35^	F	61	5000	5000	N/A	12.5	0.6
Case 13 Guevara *et al.*^35^	F	67	5000	5000	N/A	24.7	0.6
Case 14 Ranasinghe *et al.*^36^	M	73	Unknown	5000	65.3	31.0	0.9
Case 15 Ranasinghe *et al.*^36^	F	66	Unknown	5000	62.1	30.0	0.9
Case 16 Ranasinghe *et al.*^36^	M	79	N/A	5000	46.5	32.2	0.9

bd, bis in die (twice a day); F, Female; IU, international units; IVT, intravenous thrombolysis; M, male; N/A, not available; NIHSS, National institutes of Health Stroke Scale; PTT, prothrombin time; s.c., subcutaneous; y.o, years old.

In the systematic literature review, a total of 34 articles were identified and 7 articles including five case reports (Fontaine *et al.*,^31^ Bereczki *et al.*,^32^ Safouris *et al.*,^33^ Danoun *et al.*,^34^ and Warner *et al.*^12^) and two case series of three patients each (Guevara *et al.*,^35^ Ranasinghe *et al.*^36^) were retained (see Figure 1). These 11 patients [mean age ± SD 68.2 ± 8.5 years, 45.5% female, pmRS 2 (IQR 2, 5)] were analyzed. Heparin was given in 10 of 11 patients, 1 patient received enoxaparin. No adverse reactions associated with the use of protamine were reported.

To sum up, 16 patients received IVT after antagonization of heparin with protamine [65.2 ± 13.1 years, 37.5% female, median pmRS 1 (1, 4)] These patients were compared to 31 patients (PSM 1:2 ratio, match tolerance 0.2) [mean age 76.2 ± 10.9 years, 45% female, median pmRS 1 (0, 2)] (for further details see Figure 1). Median NIHSS on admission was 9 (IQR 5, 15) *versus* 7 in the control cohort (IQR 3, 12) (*p* = 0.296). Regarding the protamine group, four patients had mild (NIHSS 1–4, 18.8%), eight moderate (NIHSS 5–15, 50.0%), two moderate to severe (NIHSS 16–20, 12.5%), and two severe stroke (NIHSS 21–42, 12.5%; see Tables 1 and 2, and Table 3 for control patients). Overall, 15 of 16 patients in the protamine group (81.9%) benefited from treatment, with median NIHSS improvement of 5 points (IQR 4, 9) compared to 21 patients (67.7%) with a median NIHSS improvement of 2 points (IQR 0, 4) in the control group (*p* = 0.13). Neurological improvement was seen in 68.8% of protamine-treated patients *versus* 38.7% of control patients (*p* = 0.028). An unfavorable outcome (mRS > 2) was seen in 4 patients (25.0%) *versus* 12 patients (38.7%) (*p* = 0.576), while mortality was 6.3% *versus* 22.6% (*p* = 0.236). Good outcome at follow-up was observed in 56.3% *versus* 58.1% of patients (*p* = 0.576).

**Table 2.** Results and outcome measures of patients treated with IVT after antagonization of UFH with protamine.

Case no.	NIHSS admission	NIHSS discharge	Substantial neurological improvement	pmRS	mRS discharge	mRS in 3-month follow-up	sICH	Death	Other applicable warnings
Case 1	5	5	No	0	3	2	No	No	Recent vascular (arterial) puncture, severe hypertension
Case 2	5	0	Yes	4	4	3	No	No	None
Case 3	4	0	Yes	1	1	0	No	No	None
Case 4	5	1	Yes	0	1	0	No	No	None
Case 5	4	0	Yes	1	1	0	No	No	None
Case 6 Fontaine *et al.*^31^	12	5	Yes	2	5	3	No	No	Recent vascular (arterial) puncture
Case 7 Bereczki *et al.*^32^	9	3	No	N/A	1	N/A	No	No	Recent vascular (arterial) puncture
Case 8 Warner *et al.*^12^	4	0	Yes	2	2	N/A	No	No	Recent vascular (arterial) puncture
Case 9 Safouris *et al.*^33^	26	11	Yes	3	5	3	No	No	None
Case 10 Danoun *et al.*^34^	16	0	Yes	N/A	0	0	No	No	None
Case 11 Guevara *et al.*^35^	12	0	Yes	N/A	1	0	No	No	Recent vascular (arterial) puncture
Case 12 Guevara *et al.*^35^	11	1	Yes	N/A	1	1	No	No	Recent vascular (arterial) puncture
Case 13 Guevara *et al.*^35^	8	2	No	N/A	1	1	No	No	None
Case 14 Ranasinghe *et al.*^36^	6	0	Yes	1	2	2	No	No	None
Case 15 Ranasinghe *et al.*^36^	18	N/A	N/A	N/A	N/A	N/A	No	No	None
Case 16 Ranasinghe *et al.*^36^	21	N/A	N/A	N/A	6	6	Yes	Yes, after MT	None

IVT, intravenous thrombolysis; MT, mechanical thrombectomy; N/A, not available; NIHSS, National Institutes of Health Stroke Scale; (p)mRS, (premorbid) modified Rankin Scale; sICH, symptomatic intracerebral hemorrhage.

**Table 3.** Propensity score-matched patients treated with IVT (1:2 ratio, match tolerance 0.2): baseline characteristics, results, and outcome measures.

Control patient no.	Sex	Age (in years)	NIHSS admission	NIHSS discharge	Substantial neurological improvement	pmRS	mRS discharge	mRS at 3 months	sICH	death
1	F	69	22	N/A	N/A	4	6	6	No	Yes
2	M	82	9	9	No	4	5	5	No	No
3	M	52	4	3	No	0	3	N/A	No	No
4	M	79	12	8	No	3	3	3	No	No
5	F	90	9	N/A	N/A	4	6	6	No	Yes
6	F	73	1	0	Yes	0	0	0	No	No
7	F	90	7	3	No	0	2	0	No	No
8	F	85	12	N/A	N/A	1	6	6	No	Yes
9	F	64	35	2	Yes	0	2	6	No	No
10	M	65	13	0	Yes	0	1	0	No	No
11	F	77	4	1	Yes	1	1	3	No	No
12	M	62	9	7	No	0	3	3	No	No
13	F	76	9	6	No	0	3	2	No	No
14	M	73	1	0	Yes	0	0	0	No	No
15	F	81	5	0	Yes	1	2	1	No	No
16	M	50	5	5	No	0	2	1	No	No
17	F	92	21	15	No	4	5	6	No	No
18	F	84	5	0	Yes	0	0	0	No	No
19	F	81	12	12	No	0	5	2	No	No
20	M	77	3	1	Yes	1	2	2	No	No
21	M	57	2	2	No	0	1	2	No	No
22	M	63	8	4	Ni	0	2	1	No	No
23	F	82	2	0	Yes	1	1	1	No	No
24	M	81	3	8	No	0	5	5	No	No
25	F	84	8	0	Yes	1	1	6	No	No
26	M	80	1	0	Yes	0	0	0	No	No
27	M	86	14	13	No	3	5	6	No	No
28	M	70	7	2	No	0	3	2	No	No
29	M	83	2	2	No	0	1	1	No	No
30	M	81	3	0	Yes	3	3	0	No	No
31	M	84	1	1	No	2	4	2	No	No

F, Female; IVT, intravenous thrombolysis; M, Male; N/A, not available; NIHSS, National Institutes of Health Stroke Scale; (p)mRS, (premorbid) modified Rankin Scale; sICH, symptomatic intracerebral hemorrhage.

## Discussion

So far, the safety and efficacy of IVT after heparin antagonization with protamine has not been evaluated in large studies. In our case series and systematic review of the published cases of IVT in heparin-/protamine-treated AIS patients published from 2011 until the end of September 2021,^12,31–36^ the main findings were as follows.

First, IVT following heparin antagonization with protamine seems to be safe and effective. The protamine-treated patients seem to benefit from IVT treatment. Substantial neurological improvement was seen in 68.8% of protamine-treated patients. Overall, 15 of 16 cases showed an improved NIHSS on discharge compared to the NIHSS prior to IVT. Good clinical outcome at follow-up was observed in 56.3% of protamine-treated patients. None of our patients developed hemorrhagic transformation or (s)ICH after IVT following heparin antagonization with protamine. In the literature review, only one patient suffered sICH – however, this was in a patient with clinical deterioration and secondary large vessel occlusion who then received endovasular therapy (EVT). Compared to the control group, there were no differences regarding unfavorable outcome nor mortality.

All in all, no increase in total incidence of sICH rate has been reported in our cohort in direct comparison to pivotal trials on IVT, such as the National Institute of Neurological Disorders and Stroke rtPA Stroke Study Group (NINDS) study, the Alteplase Thrombolysis for Acute Noninterventional Therapy in Ischemic Stroke (ATLANTIS) study, or the European Cooperative Acute Stroke Study (ECASS) study.^37–39^ A study conducted by Brunner *et al.*^37^ suggested that microbleeds are already present in patients who experience sICH after IVT. However, this is true for all patients treated with IVT, yet IVT is generally performed without a mandatory MRI to visualize microbleeds.

Our study showed no adverse events or side effects due to protamine, including type I anaphylactic reactions, such as bronchospasm, hypotension, pulmonary hypertension, pulmonary vasoconstriction, and bronchoconstriction. Such side effects are experienced in 0.06–10.6% of all protamine administrations and are commonly associated with preexisting food allergies, specifically fish allergies or rapid administration of protamine.^40–42^ In particular, none of our patients experienced thrombosis after antagonization of heparin. Overall, it appears that protamine administration does not increase the risk of thrombosis.^43^

Except for one patient, all of our patients received a full-dose IVT (0.9 mg rtPA/kg body weight). Regarding the case reports and series, except for the case series by Guevara *et al.*^35^ and the case report of Warner *et al.*,^12^ full dose of IVT has also been administered. The authors discussed the dosage reduction to 0.6 mg rtPA/kg body weight with the aim to reduce the risk of sICH.^12,35,44^ Overall, a noninferiority of effectiveness has been described in literature and a reduced IVT dosage can be used in a specific collective of patients but is not usually recommended.^1,9^

In our described cases, aPTT was usually checked again after antagonization of heparin with protamine and before IVT administration. According to treatment guidelines, an aPTT > 40 s is a contraindication for IVT; however, thrombolytic therapy should not be delayed while results are pending unless in patients under coagulation, for example, heparin. Nonetheless, there are also case reports on IVT despite significantly elevated aPTT values without complications.^45,46^ In summary, however, when antagonizing heparin with protamine, a further delay of IVT due to repeated laboratory aPTT control may not be necessary.

Our study has several limitations. Information on pmRS was missing in the cases of the literature review. Moreover, the 90-day follow-up was insufficient in our case series (missing values 18.8%). Regarding the PSM analysis to compare protamine-treated patients *versus* IVT-treated patients without heparin antagonization, there was a significant difference regarding the age of the two groups. This was, on the one hand, caused by the match tolerance of 0.2 that has been used for PSM. Furthermore, only patients who were treated with IVT within the time window of 4.5 h after stroke symptom onset without any further absolute or relative contraindications for IVT were used for matching and then matched by age, sex, and NIHSS. Furthermore, we are lacking a control group as our study described only observational data.

In conclusion, our case series and mini review of all published cases of heparin-treated patients, who received protamine as an antidote, showed that IVT in these patients seems feasible, safe, and may be effective. Also, in our 2:1 PSM analysis, no significant difference was disclosed between the comparator and protamine treatment cohort. Therefore, we hypothesize that heparin antagonization with protamine may help to extend the number of AIS patients who can benefit from IVT-mediated reperfusion therapy.

Taking together, further prospective registry trials analyzing real-world data would be helpful to investigate the clinical applicability of IVT after heparin antagonization and to assess the risk of IVT treatment regarding adverse events and sICH specifically, and the functional outcome of these patients.

## Declarations

**Ethical approval and consent to participate:** This study was approved by the Local Ethics Committee (Ethics Committee at the Medical Faculty of the Eberhard Karls University and at the University Hospital Tübingen, Project No. 189/2019B02). Due to a retrospective fully anonymized analysis of the patient data, informed consent was not necessary to be obtained according to the local ethics guidelines.

**Consent for publication:** Due to a retrospective fully anonymized analysis of the patient data, consent for publication by the patients was not necessary to be obtained according to the local ethics guidelines.

**Availability of data and materials:** The data that support the findings of this study are available from the corresponding author upon reasonable request.
